# Optimal control strategy for the effects of hard water consumption on kidney-related diseases

**DOI:** 10.1186/s13104-020-05043-z

**Published:** 2020-04-06

**Authors:** Meksianis Z. Ndii, Fransiska R. Berkanis, David Tambaru, Maria Lobo, Ariyanto, Bertha S. Djahi

**Affiliations:** 1 Department of Mathematics, Faculty of Sciences and Engineering, University of Nusa Cendana, Kupang-NTT, Indonesia; 2 Department of Chemistry, Faculty of Sciences and Engineering, University of Nusa Cendana, Kupang-NTT, Indonesia; 3 Department of Computer Science, Faculty of Sciences and Engineering, University of Nusa Cendana, Kupang-NTT, Indonesia

**Keywords:** Hard water, Mathematical model, Optimal control, Sensitivity analysis, Kidney

## Abstract

**Objectives:**

We study the optimal control strategy for the effects of hard water consumption on kidney-related diseases. The mathematical model has been formulated and studied to gain insights on the optimal control strategy on the effects of hard-water consumption on kidney-related diseases. The positivity and boundedness of the solutions are determined. A global sensitivity analysis has been performed and the numerical solutions have been carried out.

**Results:**

A global sensitivity analysis shows that the control on water is an important parameter. This can reduce the proportion of individuals with kidney-dysfunction and hence reduces the proportion of individuals with kidney-related diseases. Furthermore, the numerical solutions show that with the optimal control, the proportion of individuals with kidney-related diseases can be minimised.

## Introduction

Water is an essential need of human. The increasing population and industrialization result in the contamination of water which can be a source of infectious and noninfectious diseases. Water-borne diseases are one of the major problems in the world and contribute to 2 million deaths annually [1].

Water is a pure substance consisting of hydrogen and oxygen. However, water is rarely found in its pure form. It contains the other ions. Hard-water is the water containing higher level of calcium and magnesium [2]. A long-term consumption of hard water leads to the kidney dysfunction which can cause the other-related diseases [2, 3]. As its vital function is to excrete toxins and the overload amount of any substances in the human body [4, 5], the dysfunction kidneys can be considered as a silent killer [3] and is a major problem in most developing countries [6]. In Nusa Tenggara Timur (NTT), Indonesia, the water sources, which are generally consumed by the majority of citizens, mostly contain higher concentrations of calcium and magnesium ions [7]. This might be one of the underlying factors contributing to a higher number of individuals with kidney problems [8]. Data showed that NTT is in the top four province in Indonesia with a higher number of individuals with kidney problems [8].

Mathematical models are commonly used to understand the complex phenomena [9–11]. Although a number of mathematical models have been developed to understand the transmission of water-borne diseases such as cholera and typhoid [12, 13], only small number of mathematical models of kidney-related diseases have been developed [14–18]. Tambaru et al. [7] formulated a mathematical model for kidney dysfunction and found that the control on water can reduce the number of individuals with kidney dysfunction. The model includes a control parameter on water only. Furthermore, the control parameter did not depend on time. Ndii et al. [14, 15] formulated a mathematical model for the effects of hard water consumption on kidney dysfunctions and kidney-related diseases and formulated a numerical scheme to solve the model. However, they did not include the effects of treatments/controls on the model. Walk et al. [16] developed a model to predict how myeloma cells collectively behave and proteins that involved in kidney damage. This research focuses on the interaction between cells in the proximal tubule of the kidney, free light chains, renal fibroblasts, and myeloma cells. Other mathematical models related to kidney diseases were kidney failure diagnostics model using the Artificial Neural Network (ANN) [17] and the effects of proliferative kidney diseases model using the differential equations [18]. Although these studies investigated the kidney problems but the effects of hard water consumption on kidney problems and its controls are not well studied. To the best of our knowledge, little modeling research has been conducted to investigate the effects of the consumption of hard water on the kidney-related diseases. Motivated by this, in this paper, we analyse the problem by the use of a mathematical model. In this paper, the effects of control and a global sensitivity analysis have been analysed.

## Main text

### Methods and results

#### Formulation of mathematical model

This section presents formulation of a mathematical model of the effects of hard water consumption on kidney-related diseases. We extend the model of Tambaru et. al. [7] and Ndii et al. [14] by including the kidney-related disease compartment and the control parameters. Let *S* be susceptible class, *I* be kidney dysfunction class, $I_R$ be the kidney-related diseases class, and *R* be recovered class. Individuals experience kidney dysfunction when they consume hard water at a rate $\beta \lambda (W)$. After a certain period, they attract the kidney-related disease at a rate $\gamma$. Individuals with kidney dysfunctions recover after they undergo the treatment at a rate $p_1u_1$ where $p_1$ is the recovery probability due to treatment and $u_1$ is the treatment/control rate. The individuals with kidney related diseases recover due to self-recovery at a rate $\tau$ and treatment/control at a rate $p_2u_2$ where $p_2$ is the recovery probability due to treatment and $u_2$ is the treatment/control rate. An increase level of the hardness of water is determined by the parameter *b* and limited by the carrying capacity, K. The level of hardness of water decreases when the control on water is implemented at a rate $u_3$. The model is governed by the following system of differential equations

1 $$\begin{aligned} \begin{aligned} \frac{dS}{dt}&=A-\beta \lambda (W)S-\mu S,\\ \frac{dI}{dt}&=\beta \lambda (W)S -\gamma I -\mu I-p_1u_1(t)I,\\ \frac{dI_R}{dt}&=\gamma I-\tau I_R -\mu I_R-p_2u_2(t)I_R,\\ \frac{dR}{dt}&=\tau I_R +p_1u_1(t)I+p_2u_2(t)I_R- \mu R,\\ \frac{dW}{dt}&=bW\left( 1-\frac{W}{K}\right) - u_3 W. \end{aligned} \end{aligned}$$

The $\lambda (W)$ is the probability of individuals attracting kidney dysfunction which depends on the concentrations of calcium and magnesium in the water which is governed by the following equation,

2 $$\begin{aligned} \lambda (W)=\frac{W}{K+W}. \end{aligned}$$

It is clear that the maximum probability of catching kidney dysfunction is set to be 0.5 and therefore, the maximum concentrations of calcium and magnesium in the water is equal to carrying capacity, *K*. The *A* is $\mu N$ where *N* is the total human population. We assume a constant human population. The control parameters ($u_1(t)$, $u_2(t)$, $u_3(t)$) are functions of time. Furthermore, all parameters are positive and the initial conditions are given by: $S(0)>0$, $I(0)>0$, $I_R(0)>0$, $W(0)>0$.

The parameter descriptions, ranges of values, references, and units are given in Additional file 1: Table S1. This is used in the numerical simulation and sensitivity analysis.

### Analysis of the model

#### Non-dimensionalization of the model

In this section, we nondimensionalised the model. To make system (1) dimensionless, we made the following substitution: $S=sN$, $I=iN$, $I_R=i_rN$, $R=rN$, and $W=wK$. We obtain the following system of equation

3 $$\begin{aligned} \begin{aligned} \frac{ds}{dt}&=\mu -\beta \lambda (w)s-\mu s,\\ \frac{di}{dt}&=\beta \lambda (w)s -\gamma i -\mu i-p_1u_1(t)i,\\ \frac{di_r}{dt}&=\gamma i-\tau i_r -\mu i_r-p_2u_2(t)i_r,\\ \frac{dr}{dt}&=\tau i_r +p_1u_1(t)i+p_2u_2(t)i_r- \mu r,\\ \frac{dw}{dt}&=bw\left( 1-w\right) - u_3(t) w. \end{aligned} \end{aligned}$$

where

4 $$\begin{aligned} \lambda (w)=\frac{w}{1+w}. \end{aligned}$$

### Positivity and boundedness of the solution

This section presents the positivity and boundedness of the mathematical model. The model describes the changes in human population and hence it is well-posed if it satisfies the positivity and boundedness conditions.

#### **Theorem 1**

*Given that the initial conditions of system are positive, the solutionss*(*t*), *i*(*t*), $i_r(t)$*andw*(*t*) *are nonnegative for all*$t>0$.

#### *Proof*

Assume that $T=\sup \{t>0, s>0, i>0, i_r>0, w>0 \} \in (0, t]$. Clearly, $T>0$. From the first equation of the Eq. (3), we obtain

5 $$\begin{aligned} \begin{aligned} \frac{d}{dt}\left( s\exp \left( \int _{0}^{t}\beta \lambda (W)(u)du+\mu t \right) \right) =a\exp \left( \int _{0}^{t}\beta \lambda (W)(u)du+\mu t \right) . \end{aligned} \end{aligned}$$

By integrating (5) from 0 to *T* to obtain

$$\begin{aligned} &s(T)\exp \left( {\int_0^t {\beta \lambda (W)(u)du + \mu t} } \right) - s(0) = \int_0^T a \exp \left( {\int_0^t {\beta \lambda (W)(v)dv + \mu u} } \right)du,\\&s(T) = s(0)\exp \left\{ { - \left( {\int_0^t \beta \lambda (W)(u)du + \mu t} \right)} \right\} + \exp \left\{ { - \left( {\int_0^t \beta \lambda (W)(u)du + \mu t} \right)} \right\} \\ &\qquad \int_0^T a \exp \left( {\int_0^t \beta \lambda (W)(v)dv + \mu u} \right)du > 0. \end{aligned}$$

Similarly, it can be shown for $i(t)>0$, $i_r(t)>0$, $r(t)>0$, and $w(t)>0$ for all $t>0$. $\square$

#### **Theorem 2**

*Let*

6 $$\begin{aligned} \Omega _H= & {} \left\{ (s, i, i_r, r)\in (\mathbb {R}_0^+)^4 | 0\le s(t)+i(t)+i_r(t)+r(t)\le 1\right\} , \end{aligned}$$

7 $$\begin{aligned} \Omega _W= & {} \left\{ w\in \mathbb {R}_0^+ | 0\le w(t)\le 1-\frac{u_3}{b}\right\} . \end{aligned}$$

*Define*$\Omega =\Omega _H \times \Omega _B$. *If*$N(0)\le 1$*and*$w(0)\le 1-\frac{u_3}{b}$, *then the region*$\Omega$*is positively invariant for Model* (3) *for non-negative initial conditions.*

#### *Proof*

Let $N=s+i+i_r+r$. Then we obtain

$$\begin{aligned} \frac{dN}{dt}=\frac{ds}{dt}+\frac{di}{dt}+\frac{di_r}{dt}+\frac{dw}{dt}=\mu -\mu N(t). \end{aligned}$$

Assuming that $N(0)\le 1$, we conclude that $N(t)\le 1$. Therefore, the Eq. (6) defines the biologically feasible region for the human population. For the concentrations of calcium and magnesium in the water, it follows that

$$\begin{aligned} \frac{dw}{dt}=bw\left( 1-w\right) - u_3 w \le bw-b-u_3. \end{aligned}$$

If $w(0)\le 1-\frac{u_3}{b}$, then $w(t)\le 1-\frac{u_3}{b}$. Therefore, Eq. (7) defines the biologically feasible region for the concentration of calcium and magnesium in the water. From Eqs. (6) and (7), we know that *N*(*t*) and *w*(*t*) are bounded for all $t>0$. Therefore, every solution of Model (3) with initial condition in $\Omega$ remains in $\Omega$. $\square$

### Sensitivity analysis

In sensitivity analysis, we use the combination of Latin hypercube sampling (LHS) and Partial rank correlation coefficient (PRCC) multivariate analysis to determine the most influential parameters of the model [19]. First, we measure against the increasing proportion of individuals with kidney dysfunction which is

8 $$\begin{aligned} C_I=\int _{0}^{T}\beta \lambda (w)s(t)dt. \end{aligned}$$

The results of sensitivity analysis are given in Additional file 1: Figure S1.

Additional file 1: Figure S1 shows that the parameters $\beta$, *b*, $u_3$ are the most influential parameters. The first two have a positive relationship and the last one has a negative relationship.

We measure against the increasing proportion of individuals with kidney-related diseases, which is

9 $$\begin{aligned} C_{IR}=\int _{0}^{T}\gamma i(t) dt. \end{aligned}$$

**Fig. 1:**
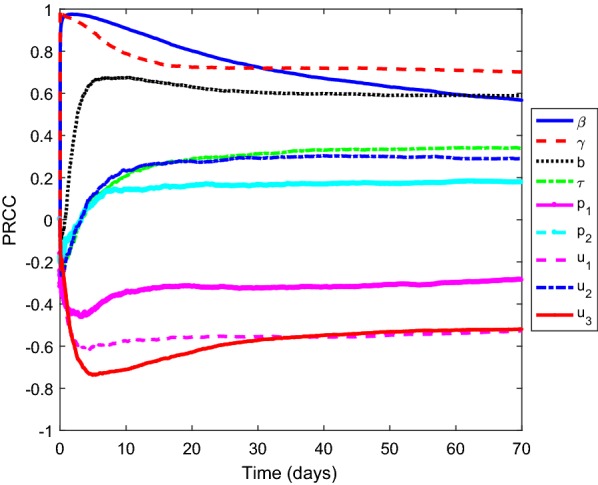
PRCC values when measured against the increasing number of individuals with kidney-related diseases

Figure 1 presents the results of a sensitivity analysis when measured against the increasing proportion of individuals with kidney-related diseases. It shows that the parameter $\gamma$, $\beta$, $b\,u_1$ and $u_3$ are the most influential parameters where the first three have the positive relationship and the latter has negative relationship.

### Optimal control analysis

In an optimal control approach, we define the objective functional as follows

10 $$\begin{aligned} J(u_1, u_2, u_3)=\int _{0}^{T} (\zeta _1i+\zeta _2i_r+\zeta _3 w +\zeta _4 u_1^2 +\zeta _5 u_2^2 +\zeta _6 u_3^2) dt. \end{aligned}$$

The $\zeta _1$, ..., $\zeta _6$ are the weight constants for individuals with kidney dysfunction, kidney-related diseases, the concentrations of calcium and magnesium in the water, the cost of control on *i*, the cost of control on $i_r$, and the cost of control on *w*, respectively. The necessary conditions that an optimal control have to satisfy are based on the Pontryagin’s Maximum Principle [20]. The Hamiltonian function is the following

11 $$\begin{aligned} \begin{aligned} H&=(\zeta _1i+\zeta _2i_r+\zeta _3 w +\zeta _4 u_1^2 +\zeta _5 u_2^2 +\zeta _6 u_6^2)\\&\quad +\lambda _s\left( \mu -\beta \lambda (w)s-\mu s\right) \\&\quad +\lambda _i\left( \beta \lambda (w)s -\gamma i -\mu i-p_1u_1i\right) \\&\quad +\lambda _{i_r}\left( \gamma i-\tau i_r -\mu i_r-p_2u_2i_r\right) \\&\quad +\lambda _r\left( \tau i_r - \mu r\right) \\&\quad +\lambda _w\left( bw\left( 1-w\right) - u_3 w\right) \end{aligned} \end{aligned}$$

#### **Theorem 3**

*Given optimal controls*$(u_1, u_2, u_3)$*and the solutions of the state*$(s^*, i^*, i_r^*, w^*)$*which minimises*$J(u_1, u_2, u_3)$*overU*. *There exist adjoint variables*$\lambda _s$, $\lambda _i$, $\lambda _{i_r}$, $\lambda _r$, $\lambda _w$*satisfying*

$$\begin{aligned} \frac{d\lambda _l}{dt}=-\frac{dH}{dl} \end{aligned}$$

*with transversality condition*$\lambda _l(t_f)=0$, *where*$l=(s, i, i_r, r, w)$. *The optimality condition is given by*

$$\begin{aligned} \frac{\partial H}{\partial u_j}=0\quad where \quad j=1,2,3. \end{aligned}$$

*The controls*$(u_1, u_2, u_3)$*are given by*

12 $$\begin{aligned} \begin{aligned} u_1^*(t)&=\min \left\{ 1,\max \left[ 0,-\frac{1}{2}\frac{p_1i(\lambda _R-\lambda _i)}{\zeta _4}\right] \right\} ,\\ u_2^*(t)&=\min \left\{ 1,\max \left[ 0,-\frac{1}{2}\frac{p_2i_r(\lambda _r-\lambda _{i_r})}{\zeta _5}\right] \right\} ,\\ u_3^*(t)&=\min \left\{ 1,\max \left[ 0,\frac{1}{2}\frac{\lambda _w w}{\zeta _6}\right] \right\} . \end{aligned} \end{aligned}$$

#### *Proof*

The differential equations governing the adjoint variables are obtained by differentiating the hamiltonian function in respect to state variables. The adjoint variables are

$$\begin{aligned} \begin{aligned} \frac{d\lambda _{S}}{dt}&=-\lambda _s\left( -\frac{\beta w}{1+w}-\mu \right) -\lambda _i\frac{\beta w}{1+w},\\ \frac{d\lambda _{i}}{dt}&=-\zeta _1-\lambda _r p_1u_1-\lambda _i(-p_1u_1-\gamma -\mu )-\lambda _{i_r}\gamma ,\\ \frac{d\lambda _{i_r}}{dt}&=-\zeta _2-\lambda _r(p_2u_2+\tau )-\lambda _{i_r}(-p_2u_2-\mu -\tau ),\\ \frac{d\lambda _{r}}{dt}&=\lambda _r\mu ,\\ \frac{d\lambda _{w}}{dt}&=-\zeta _3-\lambda _s\left( \frac{-\beta s}{1+w}+\frac{\beta ws}{(1+w)^2}\right) -\lambda _w\left( b\left( 1-w\right) -bw-u_3\right) \\&\quad -\lambda _i\left( \frac{\beta s}{1+w}-\frac{\beta w s}{(1+w)^2}\right) . \end{aligned} \end{aligned}$$

The transversality condition $\lambda _l(t_f)=0$ where $l=s, i, i_r, r, w$. Furthermore, we differentiate the hamiltonian function in respect to control variables to obtain

$$\begin{aligned} \begin{aligned} u_1^*&=-\frac{1}{2}\frac{p_1i(\lambda _r-\lambda _i)}{\zeta _4},\\ u_2^*&=-\frac{1}{2}\frac{p_2i_r(\lambda _r-\lambda _{i_r})}{\zeta _5},\\ u_3^*&=\frac{1}{2}\frac{\lambda _w w}{\zeta _6}. \end{aligned} \end{aligned}$$

Using the bounds of the controls, we obtain the characterisation of the control as given in Eq. (12). $\square$

### Numerical simulation

This section presents numerical simulations of the model. Most parameters are strongly uncertain and hence further research needs to be conducted to obtain the precise values. In the numerical simulation, the following parameter values are used: $\mu =1/65$, $\beta =0.1$, $\gamma =1/5$, $\tau =1/5$, $p_1=0.5$, $p_2=0.5$, $b=0.05$. The parameter values are taken from literature. When individuals obtain kidney dysfunction, it takes around 1–10 years to progress to kidney-related diseases. In our simulation, we use 5 years and hence the parameter $\gamma =1/5$. The recovery probability of the controls ($p_1$, $p_2$) is assumed to be 0.5. The human lifespan in Nusa Tenggara Timur is around 65 years and hence the human death rate ($\mu _H$) is taken to be 1/65. For the weight constant, we use $\zeta _1=\zeta _2=\zeta _3=\zeta _4=\zeta _5=\zeta _6=1.0$ [21]. The values of weight constants used in the numerical simulation are only of theoretical sense to illustrate the effects control in this paper. Furthermore, the initial proportions of the population are $S_H(0)=0.9$, $I_H(0)=0.1$, $I_R(0)=0$, $R(0)=0$, $W(0)=0.1$.

**Fig. 2:**
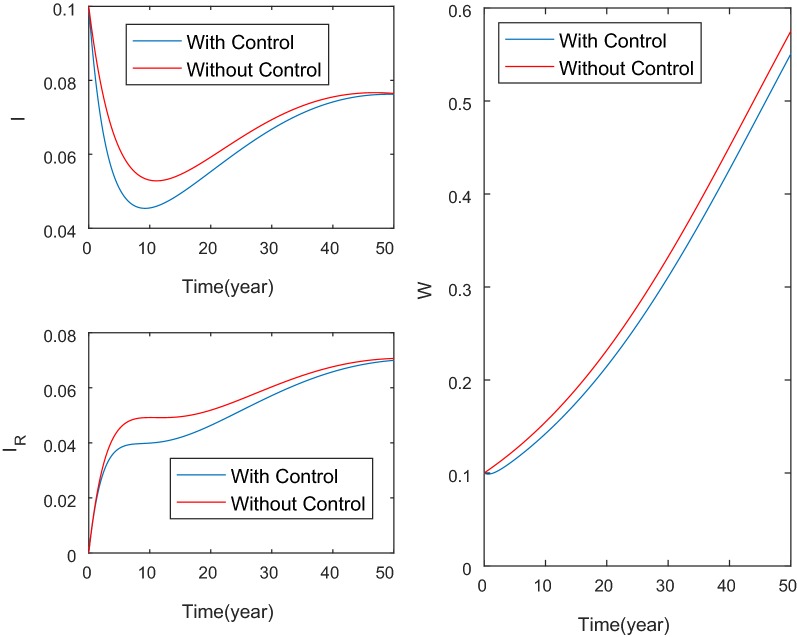
Numerical illustration of the model with and without control. The initial proportions of the population are $S_H(0)=0.9$, $I_H(0)=0.1$, $I_R(0)=0$, $R(0)=0$, $W(0)=0.1$

**Fig. 3:**
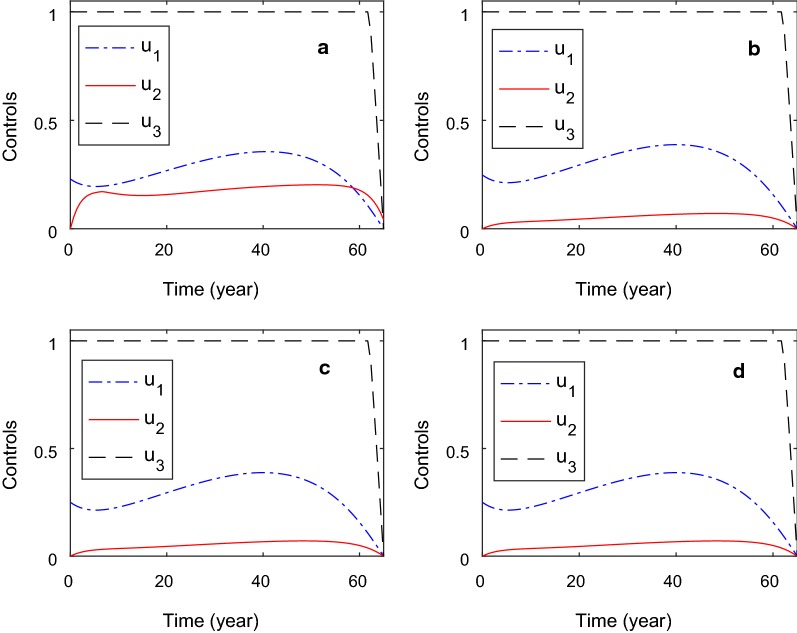
Control profile by varying the costs of control associated with kidney-related diseases ($\zeta _5$). The values of $\zeta _5$ are 0.1 (plot** a**), 1 (plot **b**), 10 (plot** c**), and 100 (plot** d**)

Figure 2 illustrates the numerical solutions of the model with and without control. The proportion of individuals with kidney dysfunction and kidney-related diseases decreases by approximately 20% and 17% respectively after the control is implemented. The control profile is given in Fig. 3b. It can be seen that the control rate on water is at highest level and decreases at the end of period.

We vary the weight $\zeta _5$ associated with the costs of control on individuals with kidney-related diseases. We use the value the weights $\zeta _5=0.1$, $\zeta _5=1$, $\zeta _5=10$, $\zeta _5=100$. The values represent very cheap, cheap, expensive and very expensive costs of controls [21]. The other weights are kept constant at the value of one. The values used in the simulation is theoretical and this is sufficient to investigate our purpose: compare the controls profile with different values of weights. The results are given in Fig. 3.

Figure 3 shows that an increase in the weights $\zeta _5$ do not significantly affects the control rate except for the $\zeta =0.1$. When the values of $\zeta _5$ is one, the control rates $u_1$ and $u_2$ are almost similar at year 6 after that the control rate $u_1$ increases and decreases at the end of the period. We also conducted the variation the weights, $\zeta _4$ and $\zeta _6$, but it gives similar results (not shown here).

### Discussion and conclusion

A global sensitivity analysis shows that the transmission rate $(\beta )$, an increase rate of the concentrations of calcium and magnesium ions in the water (*b*), and the control on the water $(u_3)$ are the most influential parameters on the increasing number of individuals with kidney dysfunction. This means that control on water aids in the minimizing the proportion of individuals with kidney dysfunction. Furthermore, the parameter $\gamma$, $\beta$, *b*, $u_1$ and $u_3$ are the most influential parameters on the increasing proportion of individuals with kidney dysfunction. This implies that an increase in the control level on individual with kidney-dysfunction and water contributes to the reduction in the proportion of individual with kidney-related diseases.

Optimal control approach has been conducted to determine the effects of control on disease dynamics. We found that around 9% reduction in the concentration of calcium and magnesium in the water leads to 20% and 17% reduction in the proportion of individuals with kidney dysfunction and kidney-related diseases. Although the costs of control associated with kidney dysfunction, kidney-related diseases and water increases (very cheap, cheap, expensive and very expensive), the results are similar. That is, the control on water is at high level followed by the control on kidney-dysfunction and kidney-related diseases. This means that the costs of controls do not significantly affect the level of control. The results are realistic since the process of transmission is on one direction: consumption of hard water resulting in kidney dysfunction and leading to kidney-related diseases. Therefore, in order to reduce the proportion of individuals with kidney-related diseases, control on water should be implemented. The results are similar to that of sensitivity analysis which shows that the water-related parameters are the important parameters.

## Limitations

Although the model provides general insights on the effects of hard water consumption on kidney related diseases, it presents a general theoretical results only. Therefore, the model can be extended to study a specific water-related diseases due to the consumption of hard water. As the parameter values are strongly uncertain, further research needs to be undertaken to obtain the precise parameter values. These are the subjects of future work.

## Supplementary information

**Additional file 1: Figure S1.** PRCC values when measured against the increasing number of individuals with kidney-dysfunction.** Table S1.** Parameter descriptions, the Parameter ranges, References, and Units.

## Data Availability

The other figure and table have been included in the additional document.

## Abbreviations

NTT: Nusa Tenggara Timur
LHS: Latin hypercube sampling
PRCC: Partial rank correlation coefficient
