# Supplementary material for: Optimal control strategy for the effects of hard water consumption on kidney-related diseases
Source: BMC Res Notes. 2020 Apr 6;13:201. doi: 10.1186/s13104-020-05043-z (PMC7137219; doi:10.1186/s13104-020-05043-z)
Supplement: Supplementary file 1 — Additional file 1: Figure S1. PRCC values when measured against the increasing number of individuals with kidney-dysfunction. Table S1. Parameter descriptions, the Parameter ranges, References, and Units. [file 13104_2020_5043_MOESM1_ESM.pdf]

Optimal Control Strategy for the Effects of Hard Water Consumption on Kidney-Related Diseases

Meksianis Z. Ndii, Fransiska R. Berkanis, David Tambaru, Maria Lobo, Ariyanto, Bertha S. Djahi

This document presents additional documents related to the paper entitled: "Optimal Control Strategy for the Effects of Hard Water Consumption on Kidney-Related Diseases"

Sensivitity Analysis

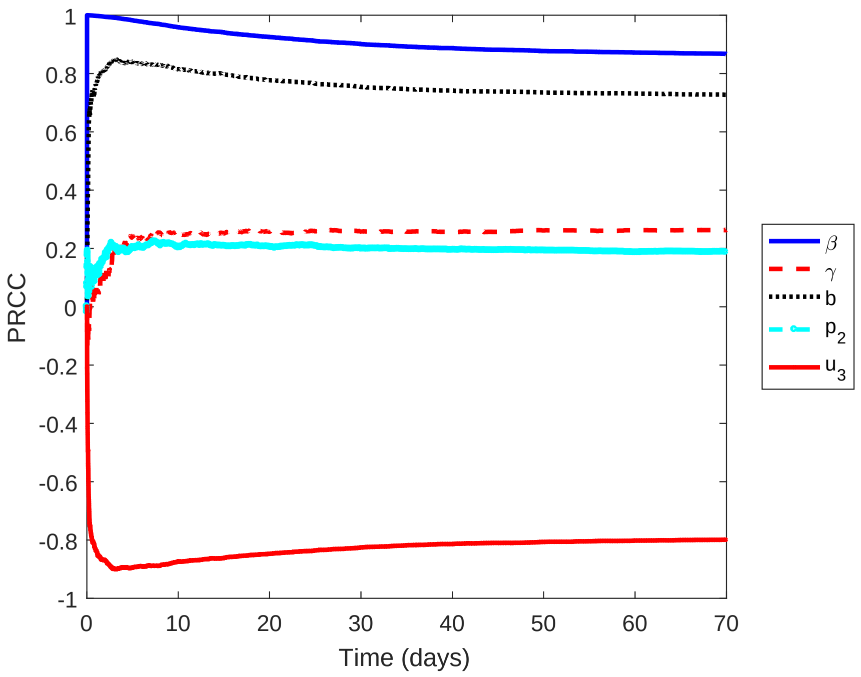

Figure S1: PRCC values when measured against the increasing number of individuals with kidney-dysfunction

Table

Table S1: Parameter descriptions, the Parameter Ranges, Refs, Units

Parameters	Description	Range	Refs.	Unit
β	Transmission probability	[0 1]	[1]	dimensionless
μ	Natural death rate	[1/80 1/60]	[2]	year^{-1}
γ	Progression rate from kidney dysfunction to kidney-related diseases	[1/5 1]	[3]	year^{-1}
u_1	Control rate on individuals with kidney dysfunction	[0 1]	[3, 4]	year^{-1}
u_2	Control rate on individuals with kidney-related diseases	[0 1]	[3, 4]	year^{-1}
p_1	Probability of recovery due to control on individuals with kidney dysfunction	[0 1]	Assumed	dimensionless
p_2	Probability of recovery due to control on individuals with kidney-related diseases	[0 1]	Assumed	dimensionless
τ	Recovery rate from kidney-related diseases	[1/10 1/2]	[3]	year^{-1}
b	Growth rate of concentration of calcium and magnesium in the water	[0 1]	[1, 3]	year^{-1}
u_3	Control rate on water	[0 1]	[1]	year^{-1}

References

[1] Tambaru D, Djahi BS, Ndii MZ. The effects of hard water consumption on kidney function: Insights from mathematical modelling. AIP Conference Proceedings. 2018;1937(1):020020.

[2] Badan Pusat Statistik Kota Kupang. Kota Kupang Dalam Angka 2018;. Online; accessed 24 February 2020. https://kupangkota.bps.go.id/publication/2018/08/16/8eca230beb6acfb1a263937d/kota-kupang-dalam-angka-2018.html.

[3] Ndii MZ, Tambaru D, Djahi BS. The effects of hard water consumption on kidney-related diseases. AIP Conference Proceedings;Accepted.

[4] Agusto FB, Leite MCA. Optimal control and cost-effective analysis of the 2017 meningitis outbreak in Nigeria. Infectious Disease Modelling. 2019;4:161 – 187. Available from: http://www.sciencedirect.com/science/article/pii/S2468042718300514.
